# Impact of lotilaner ophthalmic solution 0.25% on disease severity in patients with *Demodex* blepharitis and meibomian gland disease

**DOI:** 10.3389/fmed.2026.1719825

**Published:** 2026-01-23

**Authors:** Bridgitte Shen Lee, Elizabeth Yeu, Kavita Dhamdhere, Eric D. Donnenfeld

**Affiliations:** 1 Vision Optique, Houston, TX, United States; 2 Tarsus Pharmaceuticals, Inc., Irvine, CA, United States; 3 Virginia Eye Consultants, Norfolk, VA, United States; 4 Ophthalmic Consultants of Long Island, Long Island, NY, United States

**Keywords:** *Demodex* blepharitis with meibomian gland disease, lotilaner, meibomian gland disease severity, meibomian gland secretion score, ocular surface disease

## Abstract

**Purpose:**

To evaluate the impact of lotilaner ophthalmic solution, 0.25%, compared to vehicle, on disease severity in patients with *Demodex* blepharitis and meibomian gland disease.

**Methods:**

This post-hoc analysis pooled data from two prospective, randomized, double-masked studies evaluating lotilaner ophthalmic solution, 0.25% (Ersa, *N* = 39) and vehicle (Rhea, *N* = 40) in *Demodex* blepharitis patients with meibomian gland disease. The study utilized a modified International Workshop on Meibomian Gland Dysfunction (IWMGD) severity scale, integrating meibomian gland secretion score (MGSS) and patient-reported visual analog scale for fluctuating vision (VASFLVIS), and categorized meibomian gland disease severity as Grade 1 (subclinical), Grade 2 (mild), Grade 3 (moderate), or Grade 4 (severe). The main outcome was changes in severity grades from baseline.

**Results:**

At baseline, meibomian gland disease severity was comparable between groups. At Day 43, 47% of lotilaner-treated patients achieved Grade ≤2 (mild or better), significantly greater than 21% of vehicle-treated patients (*p* = 0.02). At Day 85, the difference increased, with 63% in the lotilaner group achieving Grade ≤2 versus 24% in the vehicle group (*p* = 0.001). For grade improvement, at Day 43, 63% of lotilaner-treated patients versus 38% of vehicle-treated patients achieved ≥1 grade improvement (*p* = 0.035); and at Day 85, 74% versus 39%, respectively, achieved ≥1 grade improvement (*p* = 0.004). No serious treatment-related adverse events were observed.

**Conclusion:**

Lotilaner ophthalmic solution, 0.25% significantly improved meibomian gland disease severity in patients with *Demodex* blepharitis and concomitant meibomian gland disease at 6 and 12 weeks compared to vehicle.

## Introduction

Meibomian gland disease is a prevalent ocular surface disorder and the leading cause of evaporative dry eye disease (DED) (1). It affects as many as 70% of Americans over the age of 60 (2), and up to 40 million people in the United States (US) (3, 4). Meibomian gland disease is characterized by chronic, diffuse abnormalities of the meibomian glands in combination with alterations to lipid and protein components of glandular secretion that compromise tear film stability and ocular surface homeostasis (5, 6). The economic burden of meibomian gland disease and its associated DED approached $4 billion annually in 2011 in the US alone (4, 7). Further, meibomian gland disease and DED have significant impacts on quality of life and visual function (5, 8, 9).

The pathophysiology of meibomian gland disease is multifactorial. Some factors include glandular obstruction, bacterial colonization, inflammatory cascade activation, and parasitic infestations (10–12). Particularly, several recent studies have evaluated the role of parasitic infections from an epidemiologic standpoint. For example, *Demodex* infestations were reported in 57–89% of meibomian gland disease cases, as compared to 34–44% of controls without meibomian gland disease (10, 13, 14). Additionally, Luo et al. reported that symptomatic meibomian gland disease occurs in approximately 90% of patients with *Demodex* blepharitis, compared to 64% in dry eye patients without *Demodex* infestation (15). This bidirectional relationship suggests a pathogenic role for *Demodex* mites in meibomian gland disease. As the cause of meibomian gland disease is multifactorial, several different treatment modalities exist for its treatment. Currently, meibomian gland disease is managed through a diverse array of techniques like lid hygiene, lid scrubs, thermal pulsation systems with gland expression, light therapy, and microblepharoexfoliation, amongst many other modalities (16–18). However, these approaches neither treat *Demodex* blepharitis nor target mite infestation in the meibomian glands.

Lotilaner ophthalmic solution, 0.25% is the first and only FDA-approved drug for treating *Demodex* blepharitis (19). As a gamma-aminobutyric acid (GABA)-gated chloride channel inhibitor selective for mites, lotilaner 0.25% directly targets the root cause of *Demodex* infestation while demonstrating a favorable safety and tolerability profile (20). Previous clinical trials have established its efficacy in reducing collarettes, curing eyelid margin erythema, and achieving mite eradication (21, 22). The objective of this analysis was to apply a modified International Workshop on Meibomian Gland Dysfunction (IWMGD)-based severity grading scale to evaluate the changes in meibomian gland disease severity following treatment with lotilaner ophthalmic solution, 0.25%, compared to vehicle, in patients with *Demodex* blepharitis and concurrent meibomian gland disease.

## Methods

### Study design and population

The current study was a post-hoc analysis of a pooled study comprising two prospective, randomized, double-masked clinical trials: Ersa (NCT05454956) and Rhea (NCT06054217) that evaluated lotilaner ophthalmic solution, 0.25% and the corresponding vehicle formulation, respectively, in patients with *Demodex* blepharitis and meibomian gland disease. Ersa enrolled 39 patients across five US clinical sites from August 2022 to May 2023, while Rhea enrolled 40 patients across two US sites from August 2023 to March 2024. The two studies shared similar designs, eligibility criteria, and sample sizes, which allowed pooling once statistical equivalence was confirmed. All studies were conducted under institutional review board-approved protocols (Advarra IRB) and adhered to the Declaration of Helsinki. All participants provided written informed consent.

### Eligibility criteria

Patients were required to meet all inclusion criteria in at least one eye: >10 upper lid lashes with collarettes, ≥1.0 mites/lash on epilated lashes from upper & lower eyelids combined, meibomian gland secretion score (MGSS) of 12–32 out of 45, Grade 1 or higher lower eyelid erythema, tear breakup time <10 seconds, ≥33% lower eyelid total gland area with intact partial to full meibomian glands on meibography, visual analog scale (VAS) score >40 for at least one patient-reported outcome (namely, fluctuating vision, itching, burning, or redness) one week prior to Day 1, and corrected distance visual acuity ≥0.7 logMAR on the Early Treatment of Diabetic Retinopathy Study (ETDRS) scale in each eye at Day 1.

Exclusion criteria included use of medications prior to Day 1 such as artificial tears (within 24 hours), antihistamines (within 30 days), cyclosporine/lifitegrast (within 60 days), any topical prostaglandin analog (within 6 months), isotretinoin (within 2 years), or other ocular/topical/systemic medications, drug delivery implants, corticosteroid, antibacterial, and/or antiparasitic treatment (within 14 days). Patients were excluded if they used any treatments for blepharitis within 14 days of Day 1, or any lid hygiene measures within 7 days of Day 1. Patients also had to be willing to forgo use of these measures for the study duration. Thermal pulsation treatments, intense pulsed light, meibomian gland probing, or therapeutic meibomian gland expression in either eye within 6 months were prohibited, as well as contact lens wear, artificial eyelashes, eyelash extensions and other cosmetic eyelashes or eyelid procedures within 7 days of enrollment. Patients with conditions or prior procedures that were deemed as confounding for study outcomes as per the opinion of the investigator were excluded, such as corneal transplant, a recent history of ocular surgery, eyelid abnormalities, ocular surface abnormality or disorder, punctal plugs, corneal disease, active ocular infection or inflammation other than blepharitis and meibomian gland disease, or systemic disease or medications known to cause dry eye. Patients with known hypersensitivities to lotilaner/any formulation components and pregnant/lactating patients were also excluded.

### Treatment regimens

In the Ersa study, patients were randomized 1:1 to receive lotilaner ophthalmic solution, 0.25% either twice daily (*n* = 21) or three times daily (*n* = 18) for 12 weeks. In the Rhea study, patients were randomized to receive vehicle with varying regimens: twice daily (*n* = 12), three times daily (*n* = 11), or three times daily for 6 weeks followed by twice daily for 6 weeks (*n* = 17).

### Assessments and severity scale

Meibomian gland assessment involved evaluation of 15 lower eyelid glands (5 consecutive glands in temporal, central, and nasal regions). Meibum quality was graded 0–3 (0 = no secretion, 1 = inspissated/toothpaste, 2 = cloudy, 3 = clear), with MGSS calculated as the sum of all 15 gland scores, yielding a score in the range of 0–45.

Patient-reported visual analog scale for fluctuating vision (VASFLVIS) was assessed using a 100-point numerical scale in which patients marked the point that best reflected their symptoms. The scale ranged from 0, indicating no discomfort, to 100, indicating maximal discomfort.

A modified IWMGD-based severity grading scale was developed by integrating both functional (MGSS) and symptomatic (VASFLVIS) parameters. The scale categorized meibomian gland disease severity into four grades: Grade 1 (subclinical): VASFLVIS <5 or MGSS >35; Grade 2 (mild): VASFLVIS ≥5 and ≤10 or MGSS ≥20 and ≤35; Grade 3 (moderate): VASFLVIS >10 and <40 or MGSS >15 and <20; and Grade 4 (severe): VASFLVIS ≥40 or MGSS ≤15 (Table 1).

**Table 1:** Meibomian gland disease severity grading algorithm.

Severity grade	VASFLVIS criteria	MGSS criteria	Clinical interpretation
Grade 1 (subclinical)	<5	>35	Minimal to no symptoms and/or functional impairment
Grade 2 (mild)	≥5 and ≤10	≥20 and ≤35	Mild symptoms and/or mild functional impairment
Grade 3 (moderate)	>10 and <40	>15 and <20	Moderate symptoms and/or moderate functional impairment
Grade 4 (severe)	≥40	≤15	Severe symptoms and/or severe functional impairment

VASFLVIS, Visual Analog Scale for Fluctuating Vision; MGSS, Meibomian Gland Secretion Score. Patients qualify for a severity grade if they meet criteria for either VASFLVIS or MGSS parameter (i.e., the most severe grade met under the VASFLVIS or MGSS criteria).

### Outcome measures

The present study measured the proportion of patients achieving meibomian gland disease severity Grade ≤2 (mild or better) and the proportion of patients demonstrating ≥1 grade improvement from baseline. Additional outcomes included changes in the individual components of the meibomian gland disease severity scale: MGSS and VASFLVIS.

### Statistical analysis

Propensity scores were calculated using logistic regression with both baseline and disease characteristics as covariates (age, collarette score, eyelid erythema, MGSS, number of glands yielding any liquid secretion, number of glands yielding clear liquid secretion, and patient-reported outcomes) to confirm data interoperability. After confirming poolability, the intention-to-treat population included all randomized subjects, with the analysis eye defined as the eye meeting all inclusion criteria, or the eye with lowest baseline MGSS if both qualified. No significant differences were observed between different dosing regimens in either study, allowing combined analysis regardless of dosing frequency.

Continuous variables were analyzed using two-sample t-tests for between-group comparisons and paired t-tests for within-group changes from baseline. Categorical variables were assessed using chi-square tests. Statistical significance was set at *p* < 0.05.

## Results

### Baseline characteristics

A total of 39 patients participated in the Ersa (lotilaner-treated) study and 40 patients participated in the Rhea (vehicle-treated) study. One patient in the Ersa group was lost to follow-up due to investigator decision, and seven patients in the Rhea group were lost to follow-up due to a non-related adverse event (*n* = 1) and patient decision (*n* = 6). Mean age was 63.7 ± 14.7 years in the lotilaner group versus 63.4 ± 12.1 years in vehicle (*p* = 0.92). Baseline MGSS (21.9 ± 5.1 vs. 22.0 ± 4.8, *p* = 0.93) and VAS for fluctuating vision (46.5 ± 31.2 vs. 51.9 ± 30.5, *p* = 0.4) were comparable between groups (Table 2).

**Table 2:** Demographic and baseline characteristics.

Characteristics	Lotilaner ophthalmic solution, 0.25% (*N* = 39)	Vehicle (*N* = 40)	*p*-value
Age, years (mean ± SD)	63.7 ± 14.7	63.4 ± 12.1	0.92
Sex, *n* (%)			
Female	23 (59)	23 (58)	1.00
Male	16 (41)	17 (43)	
Race, *n* (%)			
White	33 (84.6)	34 (85.0)	1.00
African American/Black	3 (7.7)	3 (7.5)	
Asian	3 (7.7)	3 (7.5)	
MGSS (mean ± SD)	21.9 ± 5.1	22.0 ± 4.8	0.93
VASFLVIS (mean ± SD)	46.5 ± 31.2	51.9 ± 30.5	0.4

SD = Standard Deviation; MGSS, Meibomian Gland Secretion Score; VASFLVIS, Visual Analog Scale for Fluctuating Vision.

### Meibomian gland disease severity outcomes

At baseline, meibomian gland disease severity distribution was identical between groups, with 10% of patients in each group classified as Grade ≤2 (mild or better) (*p* = 0.75).

**Figure 1:**
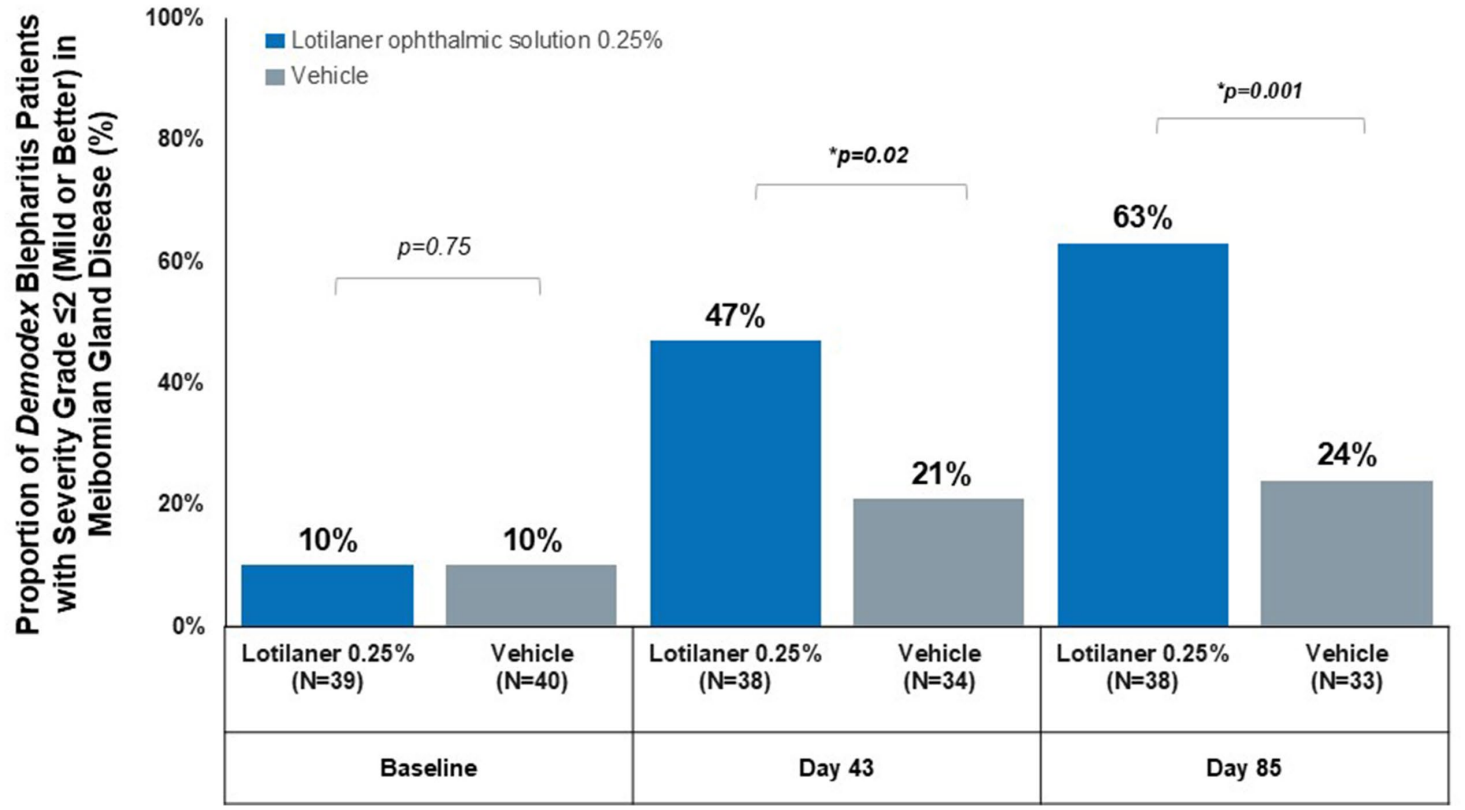
Proportion of *Demodex* blepharitis patients with severity grade ≤2 (mild or better) in meibomian gland disease.

By Day 43, 47% of lotilaner-treated patients achieved Grade ≤2 (Figure 1) compared to 21% of vehicle-treated patients (*p* = 0.02). The difference from baseline was even more apparent at Day 85, with 63% of lotilaner-treated patients achieving mild or better meibomian gland disease severity versus 24% in the vehicle group (*p* = 0.001).

**Figure 2:**
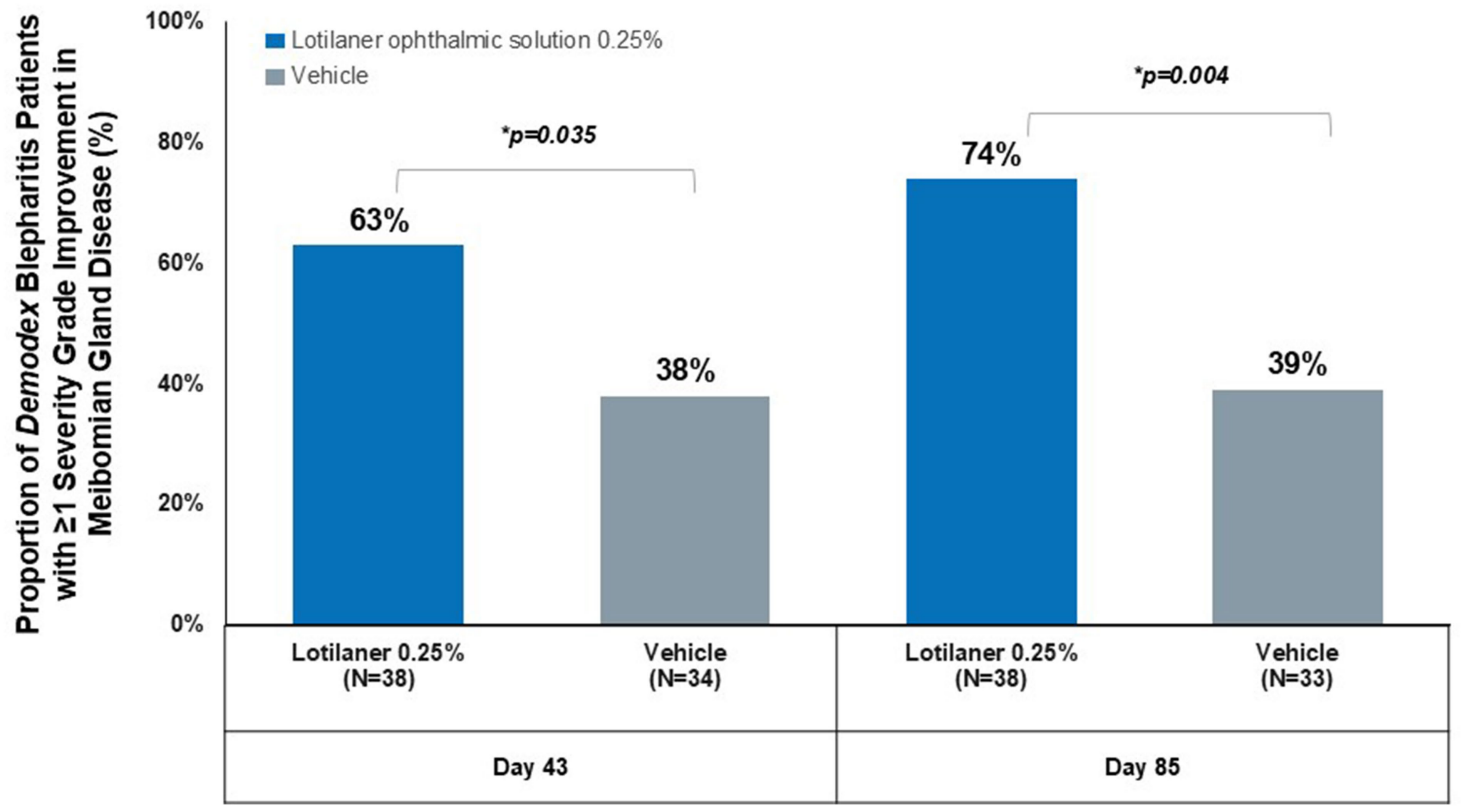
Proportion of *Demodex* blepharitis patients with ≥1 severity grade improvement in meibomian gland disease.

The proportion of patients demonstrating ≥1 grade improvement (Figure 2) was significantly higher at Day 43 (63% in lotilaner group vs. 38% in vehicle group, *p* = 0.035) and at Day 85 (74% vs. 39%, respectively; *p* = 0.004).

The lotilaner group demonstrated improvement from baseline in MGSS, improving by 26.9% at Day 43 (*Δ* + 5.7, *p* < 0.001) and 51.6% at Day 85 (Δ + 11.1, *p* < 0.001). Vehicle group changes were not statistically significant at either timepoint (*p* > 0.05). Between-group differences were statistically significant in favor of the lotilaner group at both Day 43 and Day 85 (both *p* < 0.001).

Lotilaner-treated patients also demonstrated statistically significant improvements from baseline in patient-reported fluctuating vision. Mean VASFLVIS in the lotilaner group improved by 52.3% at Day 43 (*Δ* − 22.9, *p* < 0.001) and by 71.8% at Day 85 (Δ − 32.0, *p* < 0.001). Vehicle group changes were lower at both timepoints: 22.7% at Day 43 (*p* = 0.010) and 40.7% at Day 85 (*p* < 0.001). Between-group comparisons were statistically significant in favor of the lotilaner group at Day 43 and Day 85 (both *p* < 0.01).

### Safety profile

Ocular treatment-related adverse events occurred in 2 patients (5.1%) in the lotilaner group and in 7 patients (17.5%) in the vehicle group. In the lotilaner group, there was one case of conjunctivitis (2.6%) and one case of ocular discomfort (2.6%). In the vehicle group, ocular treatment-related adverse events included one case of conjunctival irritation (2.5%), one case of dry eye (2.5%), one case of eye irritation (2.5%), one case of instillation site irritation (2.5%), two cases of noninfective conjunctivitis by one patient (2.5%), one case of ocular hyperemia (2.5%), two cases of punctate keratitis (5.0%), and one case of reduced visual acuity (2.5%). No serious treatment-related adverse events were reported in either group.

## Discussion

*Demodex* mites play a central role in the pathogenesis of meibomian gland disease through several mechanisms: mechanical blockage of gland orifices, consumption of epithelial cells and meibum, introduction of bacterial antigens, and activation of inflammatory pathways (10–12, 23). Reducing the mite burden can disrupt these processes and allow the glands to recover. The observed link between collarette grade, a marker of *Demodex* activity, and meibomian gland disease supports this mechanism (24). Treatments that directly target these mites, therefore, hold promise for improving meibum quality, gland function, and patient-reported outcomes.

This post-hoc analysis leverages a modified IWMGD-based severity grading scale to evaluate outcomes of lotilaner ophthalmic solution, 0.25% in patients with *Demodex* blepharitis and concomitant meibomian gland disease (9). The modified IWMGD-based severity grading scale incorporates both objective clinical signs and subjective patient-reported outcomes by integrating both gland function (MGSS) and patient-reported visual fluctuation (VASFLVIS). This dual-parameter approach acknowledges that meibomian gland disease severity may manifest predominantly through either functional impairment or symptomatic burden. We found that lotilaner ophthalmic solution, 0.25% not only improved individual parameters of gland function but also led to meaningful overall improvements: by 12 weeks, 74% of patients showed at least a one-grade reduction in meibomian gland disease severity.

The improvement in meibomian gland disease severity with lotilaner 0.25% over 12 weeks is an important finding. Benefits were clear at 6 weeks, and we observed an increase in magnitude at 12 weeks, suggesting that longer treatment may yield benefits. This is consistent with prior work showing that therapies such as thermal pulsation and intense pulsed light often require extended timeframes of ongoing treatment to achieve full effect in meibomian gland disease improvement (17, 25, 26). Eradicating *Demodex* mites may be an important initial step, with subsequent improvement of gland function observed after the underlying mite infestation is addressed.

The magnitude of improvement demonstrated in the lotilaner group was clinically meaningful: MGSS improved by over 50% after 12 weeks of treatment. Fluctuating vision, one of the most disabling symptoms of meibomian gland disease (27–29), improved over 70% at 12 weeks. The functional gain addresses one of the key drivers of evaporative dry eye and likely explains much of the symptom relief we observed (8, 9, 30). Safety findings were also favorable and were in line with prior literature (11, 19, 20). Overall, lotilaner ophthalmic solution, 0.25% was well-tolerated with no serious drug-related adverse events.

Limitations of this study include the small sample size and the relatively short study duration, which may not capture the full trajectory of improvement, as well as the *post hoc* pooled design. Prospective, independent studies evaluating a larger sample size may help validate the outcomes observed in this study. The study population included eyes that had at least 33% structural meibomian gland area intact and an MGSS between 12–32; the impact of more advanced gland atrophy or secretion dysfunction on recovery has yet to be determined. In addition, different dosing schedules were used in the studies. Despite this, our study possesses notable strengths including a randomized, double-masked design, consistent results across multiple outcomes, and the novel use of a clinically relevant grading scale.

In summary, lotilaner ophthalmic solution, 0.25% demonstrated statistically significant improvements in meibomian gland disease severity at 6 and 12 weeks in *Demodex* blepharitis patients with meibomian gland disease using a modified IWMGD-based severity grading scale. Lotilaner ophthalmic solution, 0.25% was well tolerated with a similar safety profile as the vehicle.

## Data Availability

The data that support the findings of this study are available from the corresponding author upon reasonable request.

## References

[1] Sheppard JD Nichols KK. Dry eye disease associated with Meibomian gland dysfunction: focus on tear film characteristics and the therapeutic landscape. Ophthalmol Ther. (2023) 12:1397–418. doi: 10.1007/s40123-023-00669-1, 36856980 PMC10164226

[2] Kim CK Carter S Kim C Shooshani T Mehta U Marshall K . Risk factors for Meibomian gland disease assessed by Meibography. Clin Ophthalmol. (2023) 17:3331–9. doi: 10.2147/OPTH.S428468, 37937186 PMC10627068

[3] Chester T Ferguson T Chester E. Localized heat treatment for meibomian gland dysfunction: a single-center retrospective analysis of efficacy over time. Optom Vis Sci. (2023) 100:625–30. doi: 10.1097/opx.0000000000002053, 37585853 PMC10637300

[4] McDonald M Patel DA Keith MS Snedecor SJ. Economic and humanistic burden of dry eye disease in Europe, North America, and Asia: a systematic literature review. Ocul Surf. (2016) 14:144–67. doi: 10.1016/j.jtos.2015.11.002, 26733111

[5] Nichols KK. The international workshop on meibomian gland dysfunction: introduction. Invest Ophthalmol Vis Sci. (2011) 52:1917–21. doi: 10.1167/iovs.10-6997, 21450912 PMC3072156

[6] Ou S Zhang L Wu Y Yang D Jiang N Mao T . Alterations in the composition of meibomian gland secretions in patients with meibomian gland dysfunction based on Raman spectroscopy. Front Med (Lausanne). (2025) 12:1717118. doi: 10.3389/fmed.2025.1717118, 41451082 PMC12728007

[7] Yu J Asche CV Fairchild CJ. The economic burden of dry eye disease in the United States: a decision tree analysis. Cornea. (2011) 30:379–87. doi: 10.1097/ICO.0b013e3181f7f363, 21045640

[8] Wei Z Liang J Cao K Wang L Baudouin C Labbé A . A multi-center study evaluating the correlation between meibomian gland dysfunction and depressive symptoms. Sci Rep. (2022) 12:443. doi: 10.1038/s41598-021-04167-x, 35013413 PMC8748897

[9] Tomlinson A Bron AJ Korb DR Amano S Paugh JR Pearce EI . The international workshop on meibomian gland dysfunction: report of the diagnosis subcommittee. Invest Ophthalmol Vis Sci. (2011) 52:2006–49. doi: 10.1167/iovs.10-6997f, 21450918 PMC3072162

[10] Cheng S Zhang M Chen H Fan W Huang Y. The correlation between the microstructure of meibomian glands and ocular Demodex infestation: a retrospective case-control study in a Chinese population. Medicine (Baltimore). (2019) 98:e15595. doi: 10.1097/MD.0000000000015595, 31083247 PMC6531270

[11] Rhee MK Yeu E Barnett M Rapuano CJ Dhaliwal DK Nichols KK . Demodex blepharitis: a comprehensive review of the disease, current management, and emerging therapies. Eye Contact Lens. (2023) 49:311–8. doi: 10.1097/ICL.0000000000001003, 37272680 PMC10351901

[12] Liu J Sheha H Tseng SC. Pathogenic role of Demodex mites in blepharitis. Curr Opin Allergy Clin Immunol. (2010) 10:505–10. doi: 10.1097/ACI.0b013e32833df9f4, 20689407 PMC2946818

[13] Lim-Bon Siong R de Venecia AB III. *Demodex* sp. infestation in anterior blepharitis, meibomian-gland dysfunction, and mixed blepharitis. Philipp J Ophthalmol. (2011) 36:15–22.

[14] Trattler W Karpecki P Rapoport Y Sadri E Schachter S Whitley WO . The prevalence of Demodex blepharitis in US eye care clinic patients as determined by Collarettes: a pathognomonic sign. Clin Ophthalmol. (2022) 16:1153–64. doi: 10.2147/OPTH.S354692, 35449733 PMC9017705

[15] Luo X Li J Chen C Tseng S Liang L. Ocular Demodicosis as a potential cause of ocular surface inflammation. Cornea. (2017) 36:S9–s14. doi: 10.1097/ICO.0000000000001361 28902017 PMC5676568

[16] Gupta PK Holland EJ Hovanesian J Loh J Jackson MA Karpecki PM . TearCare for the treatment of Meibomian gland dysfunction in adult patients with dry eye disease: a masked randomized controlled trial. Cornea. (2022) 41:417–26. doi: 10.1097/ICO.0000000000002837, 34581297 PMC8895971

[17] Lane SS DuBiner HB Epstein RJ Ernest PH Greiner JV Hardten DR . A new system, the LipiFlow, for the treatment of meibomian gland dysfunction. Cornea. (2012) 31:396–404. doi: 10.1097/ICO.0b013e318239aaea, 22222996

[18] Tauber J Owen J Bloomenstein M Hovanesian J Bullimore MA. Comparison of the iLUX and the LipiFlow for the treatment of Meibomian gland dysfunction and symptoms: a randomized clinical trial. Clin Ophthalmol. (2020) 14:405–18. doi: 10.2147/OPTH.S234008, 32103887 PMC7024784

[19] Gaddie IB Donnenfeld ED Karpecki P Vollmer P Berdy GJ Peterson JD . Lotilaner ophthalmic solution 0.25% for Demodex blepharitis: randomized, vehicle-controlled, Multicenter, phase 3 trial (Saturn-2). Ophthalmology. (2023) 130:1015–23. doi: 10.1016/j.ophtha.2023.05.030, 37285925

[20] Gonzalez-Salinas R Karpecki P Yeu E Holdbrook M Baba SN Ceballos JC . Safety and efficacy of lotilaner ophthalmic solution, 0.25% for the treatment of blepharitis due to demodex infestation: a randomized, controlled, double-masked clinical trial. Cont Lens Anterior Eye. (2022) 45:101492. doi: 10.1016/j.clae.2021.101492, 34332895

[21] Yeu E Holdbrook M Baba SN Ceballos JC Massaro-Corredor M Corredor-Ortega C . Treatment of Demodex blepharitis: a prospective, randomized, controlled, double-masked clinical trial comparing topical Lotilaner ophthalmic solution, 0.25% Eyedrops to vehicle. Ocul Immunol Inflamm. (2023) 31:1653–61. doi: 10.1080/09273948.2022.2093755, 35914297

[22] Yeu E Wirta DL Karpecki P Baba SN Holdbrook M. Lotilaner ophthalmic solution, 0.25%, for the treatment of Demodex blepharitis: results of a prospective, randomized, vehicle-controlled, double-masked, pivotal trial (Saturn-1). Cornea. (2023) 42:435–43. doi: 10.1097/ICO.0000000000003097, 35965392 PMC9973441

[23] Nicholls SG Oakley CL Tan A Vote BJ. Demodex species in human ocular disease: new clinicopathological aspects. Int Ophthalmol. (2017) 37:303–12. doi: 10.1007/s10792-016-0249-9, 27160273

[24] Yeu E Koetting C. Meibomian gland structure and function in patients with Demodex blepharitis. J Cataract Refract Surg. (2025) 51:359–65. doi: 10.1097/j.jcrs.0000000000001619, 39853246

[25] Sabeti S Kheirkhah A Yin J Dana R. Management of meibomian gland dysfunction: a review. Surv Ophthalmol. (2020) 65:205–17. doi: 10.1016/j.survophthal.2019.08.007, 31494111

[26] Tao JP Shen JF Aakalu VK Foster JA Freitag SK McCulley TJ . Thermal pulsation in the management of meibomian gland dysfunction and dry eye: a report by the American Academy of ophthalmology. Ophthalmology. (2023) 130:1336–41. doi: 10.1016/j.ophtha.2023.07.009 37642619

[27] Ajouz L Hallak J Naik R Nguyen A Zhao C Robinson MR . Evaluation of the impact of meibomian gland dysfunction using a novel patient-reported outcome instrument. J Ocul Pharmacol Ther. (2024) 40:48–56. doi: 10.1089/jop.2023.0080, 37910805 PMC10890943

[28] Suzuki T Kitazawa K Cho Y Yoshida M Okumura T Sato A . Alteration in meibum lipid composition and subjective symptoms due to aging and meibomian gland dysfunction. Ocul Surf. (2022) 26:310–7. doi: 10.1016/j.jtos.2021.10.003, 34666148

[29] Koh S. Mechanisms of visual disturbance in dry eye. Cornea. (2016) 35:S83–s8. doi: 10.1097/ico.0000000000000998, 27583799

[30] Papas EB Keay L Golebiowski B. Estimating a just-noticeable difference for ocular comfort in contact lens wearers. Invest Ophthalmol Vis Sci. (2011) 52:4390–4. doi: 10.1167/iovs.10-7051, 21498609

